# Is calprotectin a novel biomarker of neuroinflammation in diabetic periferal neuropathy?

**DOI:** 10.1186/s13098-015-0030-7

**Published:** 2015-04-24

**Authors:** Suzan Tabur, Hakan Korkmaz, Mesut Ozkaya, Sefika Nur Aksoy, Ersin Akarsu

**Affiliations:** Department of Internal Medicine, Division of Endocrinology, Gaziantep University, Faculty of Medicine, 27100 Sahinbey, Gaziantep, Turkey; Department of Clinical Biochemistry, Gaziantep University, Faculty of Medicine, 27100 Sahinbey, Gaziantep, Turkey

**Keywords:** Calprotectin, Inflammation, Diabetic peripheral neuropathy

## Abstract

**Background:**

In the present study, we aimed to investigate serum calprotectin levels in patients with diabetic peripheral neuropathy, and possible role of this molecule in the disease pathogenesis.

**Method:**

Twenty nine patients with diabetic peripheral neuropathy, 30 type 2 diabetic patients without neuropathy, and 40 healthy controls were enrolled in the study. Fasting plasma glucose (FPG), high-density lipoprotein- cholesterol (HDL-C), low-density lipoprotein- cholesterol (LDL-C), total cholesterol, triglyceride, HbA1c, calprotectin and hsCRP levels were measured in diabetic and healthy control groups.

**Results:**

Serum calprotectin and hsCRP levels were significantly higher in patients with and without neuropathy than healthy controls (p < 0.001, p = 0.017, p < 0.001 and p = 0.001, respectively). Serum calprotectin and hsCRP levels were higher in diabetics with neuropathy than the ones without (p = 0.021 and p < 0.001, respectively). The positive correlation was detected between calprotectin levels and hsCRP and HbA1c in Spearman correlation analysis (r = 0.510, p < 0.001; r = 0.437, p < 0.001 respectively). The results of multiple logistic regression analysis demonstrated the important association between neuropathy development and hsCRP and serum calprotectin levels in diabetic individuals.

**Conclusion:**

Seum calprotectin levels were increased in diabetic peripheral neuropathy. It may have a role in the pathogenesis of the disease.

**Electronic supplementary material:**

The online version of this article (doi:10.1186/s13098-015-0030-7) contains supplementary material, which is available to authorized users.

## Introduction

Diabetes is encountered as one of the most challenging healthcare problems in the 21st century, and its prevalance is higher in developing countries. While for year 2030, 170% increase is expected for developing countries, 42% increase is expected for developed countries [1]. The most commonly encountered microvascular complication of type 2 diabetes is diabetic neuropathy with the prevalence of 50-60%. Neuropathy may cause decreased nerve functions and nerve blood perfusion with persistent nerve damage. Diabetic peripheral polyneuropathy increases development of foot ulceration risk, and also it increases developmental risk of necrosis, which may cause lower extremity amputations. Diabetic peripheral polyneuropathy has significant contributions in morbidity and mortality in diabetic patients [2,3].

Although it is predicted that hyperglycemia is an important pathophysiological factor in development of diabetic neuropathy, the related mechanisms have not been totally clarified. Opinions suggesting that inflammatory processes may play a role in pathogenesis of diabetic polyneuropathy are increasing. In previous studies, it has was shown that peripheral neuropathy was associated with increased levels of proinflammatory immune mediators in patients with type 2 diabetes [4,5].

Calprotectin (myeloid related protein 8/14) is a stable heterodimer belonging to S100 protein family composed of two calcium binding cytoplasmic calgranulins which are expressed in activated human granulocyte and macrophages in inflammatory conditions. Among its functions are activations of NADPH oxidase, toll like receptors 4 (TLR4), and advanced glycation end products (AGEs) receptors, which are important signallling pathways in pathogenesis of micro- and macrovascular complications of diabetes [6-8].

In the present study, we aimed to investigate serum calprotectin levels in patients with peripheral neuropathy, and possible role of this molecule in the disease pathogenesis.

## Material and method

The present prospective study was conducted in the department of Endocrinology at Medical School of Gaziantep University. Twenty nine patients with diabetic peripheral neuropathy, 30 type 2 diabetic patients without neuropathy, and 40 healthy controls were enrolled in the study.

Patients with diabetic peripheral neuropathy had symptomatic symetric distal neuropathy (i.e., hypoactive deep tendon reflexes, reduced tactile, pinprick, and/or position sensation) with at least moderate severity of one or more of the typical symptoms (pain, burning, paresthesia, mumbness or cramps) in the lower exremites.

The diagnosis of diabetic peripheral neuropathy was made according to clinical symptoms, neurologic examination and electrophysiologic investigation. Nerve conduction studies were performed with a standart electromyography equipment.

Subjects had infectious diseases, inflammatory diseases, liver failure, malignancies, neurodegenerative diseases, renal failure, cerebrovascular diseases, medical history of serious trauma to the limbs, use of neurotoxic medication, B12 vitamin deficiency, excessive alcohol comsumpsion and smokers were excluded from the study both in study and control groups.

Age, weight, height, body mass index (BMI: body weight (kg)/height (cm)^2^), and systolic (SBP) and diastolic blood pressures (DBP) of all subjects were recorded. Blood samples were collected in the morning after an 8-hour fasting period. Serum samples were stored at −80°C until calprotectin levels were measured. Fasting plasma glucose (FPG), high-density lipoprotein- cholesterol (HDL-C), low-density lipoprotein- cholesterol (LDL-C), total cholesterol, triglyceride (TG), HbA1c, high sensitive C-reactive protein (hsCRP) levels were measured in diabetic and healthy control groups.

The concentration of serum calprotectin was determined using an enzyme-linked immunosorbent assay detection kit (Hycult biotec, HK325, Human Calprotectin ELISA kit, NLD).

FPG, HDL-C, LDL-C, total cholesterol (Total-C), TG levels were measured by electrochemiluminescence method using Cobas Integra 800 model auto-analyzer (Roche Diagnostics, Germany). HbA1c levels were measured by HPLC (high performance liquid chromatography) method using Trinity Biotech-Premier Hb9210 auto-analyzer. Standard biochemistry tubes with gel were also used for the measurement of hsCRP levels with immunonephelometry assay on Dade Behring Nephelometer II device.

The study was initiated upon obtaining approval from Ethics Committee of Medical Schol at Gaziantep University (23.06.2014/246). Informed consent was obtained from all subjects prior to the study.

### Statistical analysis

Shapiro-Wilk test was used to test continuous variables for normality. Measurements of normally distributed variables (age, LDL-C) are presented as mean ± standard deviation. Those with non-normal distributions are presented as median and interquartile range (IQR). Student’s *t*-test was used in comparison of 2 independent groups of normally distributed variables; one-way analysis of variance (ANOVA) test was used when comparing more than 2 groups; and LSD test was used for paired comparisons to identify which group the difference was caused by. For non-normally distributed variables, Mann–Whitney *U* test was used to compare 2 independent groups and Kruskall Wallis test was used to compare more than 2 independent groups. DUNN test was used for post-hoc comparisons. Spearman correlation analysis was done to identify associations between the parameters in diabetic patients. Multiple linear regression analysis was conducted to identify the variables impacting the calprotectin level. Multiple logistic regression analysis was done to evaluate the relationship between neuropathy and calprotectin, hsCRP and BMI in diabetic patients. SPSS for Windows version 15 software was used for statistical analyses. The level of significance was set at *p* ≤ 0.05.

## Results

The mean age of all 3 groups and their gender distribution were similar (*p* = 0.077 and *p* = 0.609, respectively). BMI, FPG, and HbA1c levels of diabetic patients with and without neuropathy were significantly higher than the controls (*p* = 0.001, for each). While FPG and HbA1c levels in patients with neuropathy were significantly higher than patients without neuropathy; BMI in the former group was significantly lower than in the latter group (*p <* 0.001, for each; Table 1).

**Table 1:** **Clinical and metabolic parameters of the diabetic patients with or without neuropathy and healthy control groups**

	**DNP(+)**	**DNP(−)**	**HC**	**P DNP(+)-DNP(−)**	**p DNP(+)-HC**	**P DNP(−)-HC**
Age (years)*	52.59 (±13.56)	49.83 (±12.65)	47.15 (±7.21)	0.424	0.262	0.125
Gender (M/F)	11/18	15/15	13/14	0.435	0.590	0.550
BMI (kg/m^2^)	34.80 (±11.90)	40.61 (±3.70)	26.64 (±2.26)	<0.001	0.001	0.001
SBP (mmHg)	130.00 (±20.00)	135.00 (±10.00)	120 (±15.00)	0.042	0.054	<0.001
DBP (mmHg)	80 (±20.47)	80 (±13.00)	75.00 (±10.00)	0.174	0.026	<0.001
FPG (mg/dl)	205 (±97.00)	117 (±32.00)	87 (±13.00)	<0.001	0.001	0.001
HbA1c (%)	10.10 (±3.50)	7.15 (±1.10)	5.10 (±0.50)	<0.001	0.001	0.001
Total-C (mg/dl)	180.50 (±71.00)	222.00 (±38.00)	109 (±28.00)	0.001	0.528	<0.001
HDL-C (mg/dl)	38.50 (±17.00)	48.50 (±8.00)	50.00 (±9.00)	0.003	0.002	0.183
LDL-C (mg/dl)*	115.61 (±40.09)	141.77 (±27.91)	105.26 (±25.30)	0.004	0.441	<0.001
TG (mg/dl)	214.00 (±95.00)	173.50 (±67.00)	136.00 (±39.00)	0.041	<0.001	0.001
Creatinine (mg/dl)	0.85 (±0.29)	0.86 (±0.16)	0.89 (±0.11)	0.167	0.102	0.163
Duration of DM (years)	6 (±2.00)	3 (±1.00)		0.001		
Calprotectin (ng/dl)*	8.30 (±1.48)	7.41 (±1.26)	6.18 (±1.58^)^	0.021	<0.001	0.017
hsCRP (mg/dl)	52.59 (±13.56)	2.40 (±2.34)	1.12 (±0.41)	<0.001	<0.001	0.001
Medical Treatment	11/18			0.141		
OAD (n, %)	34.80 (±11.90)	14 (46.7%)				
Insulin (n, %)	130.00 (±20.00)	5 (16.7%)				
OAD + Insulin (n, %)	80 (±20.47)	11 (36.7%)				

Body Mass Index, BMI; Fasting Blood Glucose; High density lipoprotein cholesterol, HDL-C; High sensitive C reactive protein, hsCRP; Low density lipoprotein cholesterol, LDL-C; Triglyceride, TG.

**Figure 1:**
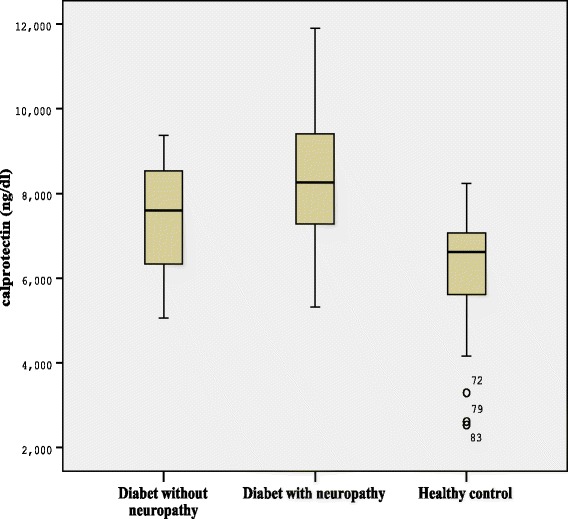
Serum concentrations of calprotectin in healthy control group and diabetic patients with or without neuropathy.

Serum calprotectin and hsCRP levels were significantly higher in patients with and without neuropathy than healthy controls (*p* < 0.001, *p* = 0.017, *p <* 0.001 and *p =* 0.001, respectively; Figure 1). Serum calprotectin and hsCRP levels were higher in diabetics with neuropathy than the ones without (*p* = 0.021 and *p <* 0.001, respectively).

While TG levels were higher in patients with neuropathy than patients without; total-C, HDL-C and LDL-C levels were determined lower (*p* = 0.041, *p* = 0.001, *p* = 0.003, *p* = 0.004, respectively). Total-C, LDL-C and TG levels were determined significantly higher in diabetic patients without neuropathy than healthy controls (*p* < 0.001, *p* < 0.001 and *p* = 0.001, respectively). TG levels in diabetic patients with neuropathy were higher than the control group, whereas HDL levels were lower (*p* < 0.001, *p* = 0.002). There was no significant difference in LDL and total-C levels between patients with neuropathy and healthy controls (*p* = 0.441, *p* = 0.528).

**Figure 2:**
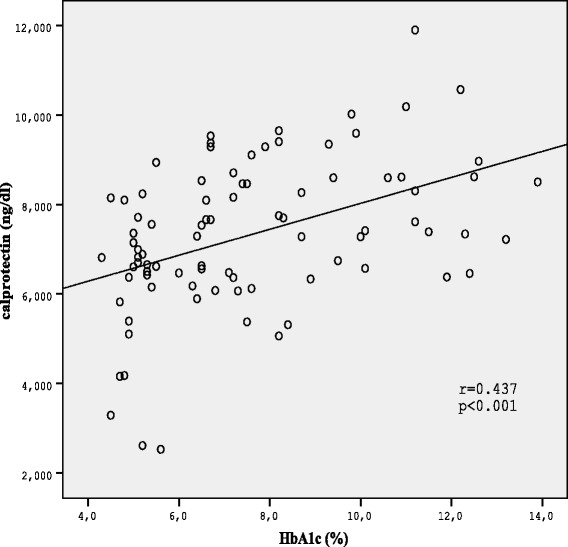
Correlation between serum calprotectin levels and HbA1C in diabetic patients.

**Figure 3:**
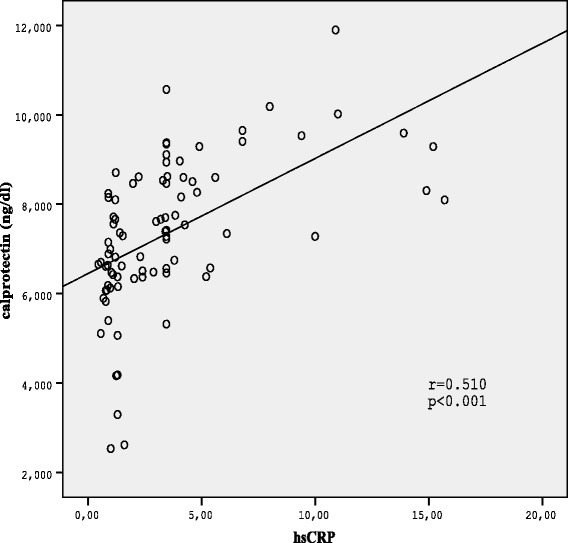
Correlation between serum calprotectin levels and hsCRP in diabetic patients.

The correlations between calprotectin and other clinical and labaratory parameters were shown in Table 2. The positive correlation was detected between calprotectin levels and hsCRP and HbA1c in Spearman correlation analysis (*r =* 0.510, *p* < 0.001;r = 0.437, *p* < 0.001 respectively; Figures 2 and 3).

**Table 2:** **Correlations between calprotectin, and other clinical and metabolic parameters in diabetic patients**

	**BMI**	**SBP**	**DBP**	**HDL-C**	**LDL-C**	**TG**	**FBP**	**HbA1C**	**hsCRP**
Calprotectin									
R	0.147	0.061	0.135	−0.166	−0.028	0.192	0.221	0.437	0.510
P	0.177	0.577	0.215	0.129	0.796	0.077	0.041	<0.001	<0.001
BMI									
R		0.237	0.146	0.097	0.126	−0.501	0.117	0.180	0.175
P		0.028	0.180	0.377	0.246	0.641	0.285	0.096	0.188
SBP									
R			0.611	0.228	0.239	−0.051	0.003	0.112	−0.030
P			<0.001	0.036	0.027	0.641	0.982	0.303	0.823
DBP									
R				0.259	0.155	−0.158	0.070	0.150	0.019
P				0.017	0.153	0.146	0.523	0.169	0.887
HDL-C									
R					0.460	0.077	0.391	0.271	−0.224
P					<0.001	0.484	<0.001	0.012	0.094
LDL-C									
R						0.171	0.224	0.070	−0.328
P						0.116	0.038	0.522	0.012
TG									
R							0.587	0.324	−0.047
P							<0.001	0.002	0.726
FBG									
R								0.729	0.207
P								<0.001	0.120
HbA1C									
R									0.203
P									0.127

Data were expressed as median (±IQR) and *mean (±SD). Mann–Whitney U and *Student’s *t*-test was used in comparison of 2 independent group. DM with neuropath group, DNP (+); DM without neuropathy group, DNP (−); Healthy control group, HC; Body Mass Index, BMI; Systolic blood pressure, SBP; Diastolic blood pressure; Fasting blood glucose; High density lipoprotein cholesterol, HDL-C; Triglyceride, TG; Low density lipoprotein cholesterol, LDL-C; High sensitive C reactive protein, hsCRP; Oral antidiabetic drug, OAD.

Multiple logistic regression analysis results demonstrated the important association between neuropathy development and hsCRP and serum calprotectin levels in diabetic individuals (Table 3).

**Table 3:** **Results of logistic regression models predicting neuropathy**

	**OR (95% Cl)**	**P value**
Calprotectin	1.771 (1.104-2.840)	0.018*
hsCRP	1.043 (1.005-1.082)	0.027*
BMI	0.976 (0.938-1.017)	0.247

*Significant at p < 0.05, High sensitive C reactive protein, hsCRP; Body Mass Index, BMI.

## Discussion

The present study is the first study describing the correlation between calprotectin and diabetic peripheral neuropathy in type 2 diabetic patients.

Calprotectin, belonging to S100 protein family, a heterodimer of two intracellular, calcium-binding proteins (S100A8 and S100A9 also referred to as MRP8 and MRP14), is critically involved in proinflamatory signalling. It is proposed that calprotectin complex is a biomarker for inflammation, and is beneficial in monitorizing disease activity. High levels of calprotectin levels are reported in chronic inflammatory diseases such as rheumatoid arthritis, allograft rejection, inflammatory bowel disease, cancer, pulmonary disease, obesity, atherosclerosis [6,9,10].

Diabetic peripheral neuropathy is chacaterized by debiliating pain, which deteriorates the lfe quality, and sensation loss. It was believed that activation of inflammatory cascade, proinflammatory cytokine upregulation, and neuroimmune communication pathways are important in structural and functional nerve damage, which caused development of diabetic peripheral neuropathy. It was proposed that AGE had an important role during this process [3,11].

The finding that AGEs stimulating signal transduction cascades through multiligand receptor the receptor for advanced glycation end products (RAGE) unveiled novel insights into diabetes and its complications. RAGE is a multi-ligand receptor interacting with certain members of S100/calgranulin family, and implicates the receptor in inflammatory disorders [12-14].

The association of Nuclear factor-kappa B (NF-κB ) activation with diabetic neuropathy has been shown in Mouse models. NF-κB is a transcription factor that is involved in the control of large number of celluler processes, such as immun and inflammatory responses. Toll-like receptors can activate NF-κB and cause an increase in expression of several inflammatory mediators. Calprotectin has also been idendifed as endogenous activator of TLR4 [11,15].

These mechanisms suggests that calprotectin may be an inflammatory marker, which has a role in pathogenesis of diabetic peripheral neuropathy.

So far, relationship between circulating levels of calprotectin and glucose homeostasis has been shown in few studies. Ortega et al. reported plasma levels of calprotectin associated with insulin resistance and low-grade inflammation in type 2 diabetic patients [16]. Schmaderer et al. adressed a correlation between calprotectin and microalbuminuria; a predictive marker of cardiovascular disease in type 2 diabetic patients [17]. Peng et al. reported that increased serum calprotectin level was associated with atherosclerosis in type 2 diabetic patients [8]. In the present study, we determined higher calprotectin levels in diabetic peripheral neuropathy, which is the most commonly encountered microvascular complication in type 2 diabetics, when compare with diabetics without neuropathy and healthy controls.

In this study calprotectin correlated significantly with hs-CRP levels supporting the role of calprotectin as an inflammation marker. Similar to our results, positive correlation was reported betwen calprotectin and hs-CRP in a few previous studies [8,18]. On the other hand there was also a positive correlation between calprotectin and HbA1c, the marker of long-term elevation of blood sugar. This result suggests that levels of glucose or glycation products may affect regulation of high calprotectin levels in diabetic complications.

In conclusion, high levels of calprotectin detected in type 2 diabetic patients with peripheral neuropathy suggest that this molecule may have a role in pathogenesis of neuroinflammation among these patients. Serum calprotectin levels in the future may be used as potential markers of its presence, severity and progression of the diabetic peripheral neuropathy. Therapeutic strategies for blocking S100A9 and its activity are recently under development in inflammatory diseases [19]. Therefore, the data from our study also support calprotectin as a drug target in diabetic peripheral neuropathy.

## Abbreviations

BMI: Body mass index
SBP: Systolic blood pressures
DBP: Diastolic blood pressures
FPG: Fasting plasma glucose
Total-C: Total cholesterol
HDL-C: High density lipoprotein- cholesterol
TG: Triglyceride
LDL-C: Low density lipoprotein-cholesterol
hsCRP: High sensitive C-reactive protein
AGE: Advanced glycation and products
TLR4: Toll like receptors 4
NF-κB: Nucleer factor- kapa B
RAGE: Receptor for advanced glycation end products
